# Experiments and Modeling on the Stain-Controlled Time- and Temperature-Dependent Cyclic Ratchetting Plasticity of the Nickel-Based Superalloy IN100

**DOI:** 10.3390/ma16051888

**Published:** 2023-02-24

**Authors:** Carl Fischer, Sophie Schackert, Thomas Seifert, Christoph Schweizer, Martin Fuchs

**Affiliations:** 1 Institute for Digital Engineering and Production (IDEeP), Offenburg University of Applied Sciences, Badstraße 24, 77652 Offenburg, Germany; 2 Fraunhofer Institute for Mechanics of Materials IWM, Wöhlerstraße 11, 79108 Freiburg, Germany

**Keywords:** cyclic loading, viscoplastic material, constitutive behavior, mechanical testing

## Abstract

In this paper, the time- and temperature-dependent cyclic ratchetting plasticity of the nickel-based alloy IN100 is experimentally investigated in strain-controlled experiments in the temperature range from 300 °C to 1050 °C. To this end, uniaxial material tests are performed with complex loading histories designed to activate phenomena as strain rate dependency, stress relaxation as well as the Bauschinger effect, cyclic hardening and softening, ratchetting and recovery from hardening. Plasticity models with different levels of complexity are presented that consider these phenomena, and a strategy is derived to determine the multitude of temperature-dependent material properties of the models in a step-by-step procedure based on sub-sets of experimental data of isothermal experiments. The models and the material properties are validated based on the results of non-isothermal experiments. A good description of the time- and temperature-dependent cyclic ratchetting plasticity of IN100 is obtained for isothermal as well as non-isothermal loading with models including ratchetting terms in the kinematic hardening law and the material properties obtained with the proposed strategy.

## 1. Introduction

Nickel-based superalloys, e.g., Haynes 230, MAR-M247 or IN100, are typically used as materials for turbine blades and nozzle guide vanes due to their high corrosion resistance and their good high-temperature strength. Since higher firing temperatures are aimed at increasing the efficiency of gas turbines, internal cooling systems for blades and vanes are designed to limit the material temperature [1]. However, spatial temperature gradients as well as the geometrical notch of cooling holes and channels result in stress and strain concentrations, so that local cyclic plastic deformations occur. Hence, those design features are preferred locations for the initiation of thermomechanical fatigue (TMF) cracks [2]. For the assessment of local stresses and strains, e.g., at cooling holes and channels in finite-element calculations of turbine blades and vanes, which are subjected to cyclic thermal and mechanical loads, as well as for the fracture mechanics assessment of TMF cracks, appropriate plasticity models and the corresponding temperature-dependent material properties of the used materials are required.

In this work, the time- and temperature-dependent cyclic ratchetting plasticity of the nickel-based superalloy IN100 is experimentally investigated and modeled under strain-controlled conditions. Therefore, time- and temperature-dependent plasticity models are reviewed in Section 1.1, and strain-controlled ratchetting is addressed in Section 1.2 before the challenge of determining temperature-dependent material properties of the plasticity models from experimental data are considered in Section 1.3. From this state of knowledge, the aims of this work are derived in Section 1.4.

### 1.1. Time- and Temperature-Dependent Cyclic Plasticity and Models

Typical phenomena occurring for materials in high-temperature applications with thermomechanical loading cycles are strain rate dependency, creep, stress relaxation and recovery from hardening, the Bauschinger effect, cyclic hardening and softening. For finite-element calculations, plenty of incremental plasticity models that account for these phenomena have been proposed, extended and applied during the last several decades. Most of them are based on the works on plastic and viscoplastic constitutive models of Chaboche, e.g., [3,4,5]. They use the kinematic hardening model of Armstrong and Frederick [6,7], which gives a nonlinear evolution equation for the backstresses and describes an exponential hardening curve using two material properties: the initial hardening modulus *C* and the dynamic recovery parameter γ that controls the decreasing hardening rate (enabling nonlinear hardening). Several backstresses are usually superimposed to obtain a good description of stress–strain hysteresis loops and hardening during reversed loading showing the Bauschinger effect. The Armstrong–Frederick kinematic hardening model was extended by a static recovery term [4] that results in a recovery from hardening with time at high temperatures occurring during thermomechanical loading. While the Armstrong–Frederick kinematic hardening law has been available for a long time as the standard material model in the finite-element programs ABAQUS and ANSYS, static recovery has been implemented in the recent versions ABAQUS/Standard (Version 2020) and ANSYS (Version 18).

For the application of the Armstrong–Frederick kinematic hardening model to non-isothermal conditions, Chaboche introduced a temperature rate term in the evolution equation that is based on thermodynamics with internal variables and accounts for a temperature-dependent hardening modulus *C* [8]. For a constant (i.e., temperature-independent) dynamic recovery parameter γ, temperature history-independent material behavior is obtained. For temperature-dependent γ, the material behaves as temperature history-dependent [4]. For an inappropriate temperature dependency of γ, unrealistic results may occur with kinks in the stress or strain history under non-isothermal conditions [9]. Consequently, a sensible temperature dependency must already be considered when determining the material property’s corresponding values. Even though this issue is solved with a temperature history-independent formulation of the Armstrong–Frederick kinematic hardening rule based on a similarity equation under temperature variation by Ohno and Wang [10,11], their approach has not prevailed. The Chaboche-type time- and temperature-dependent plasticity models were widely applied to describe the material behavior under cyclic thermomechanical loading conditions for different materials and material classes, e.g., aluminum alloys in [12,13], forged and cast steels in [14,15,16,17], nodular cast iron in [18,19], copper in [20] and nickel-based superalloys in [21,22,23,24,25,26]. Further extensions to the Armstrong–Frederick kinematic hardening law were proposed to describe better non-proportional hardening, e.g., [24,27], strain range memory, e.g., [28,29], as well as cyclic kinematic hardening and softening, e.g., [3,30].

### 1.2. Strain-Controlled Ratchetting and Ratchetting Models

The loading conditions focused on in this work are strain-controlled as relevant for cooling holes and channels where temperature gradients strongly constrain thermal strains, and TMF cracks are usually expected. The strain-controlled loading history can show mean strains induced by high initial plasticity in highly loaded regions in the first TMF cycles or by pre-straining so that ratchetting appears in the form of mean stress relaxation. Additionally, mean strains typically occur at the crack-tip even for fully reversed strain-controlled far-field loadings [31]. The Armstrong–Frederick kinematic hardening law overestimates ratchetting and, hence, mean stress relaxation under strain-controlled loading. Thus, the hardening law was extended by additional terms, which result in a slow-down of the too high ratchetting rate. These extensions were, however, primarily developed for isothermal stress-controlled cyclic loading conditions.

A recent review of cyclic plasticity models, including ratchetting terms in the kinematic hardening law, is given in [32] focusing on stress-controlled loadings. Different effects such as temperature, hardening/softening and multiaxial stress states on the ratchetting behavior are discussed. Thermomechanical fatigue loadings are, however, not addressed. A frequently used modification of the Armstrong–Frederick kinematic hardening law regarding ratchetting is the Ohno–Wang model [33], which introduces a critical state of dynamic recovery. Jiang and Sehitoglu [34] modified the Ohno–Wang model to predict a constant ratchetting rate in the case of non-proportional loading, resulting in the Jiang model. The Ohno–Wang model formulation leads to high-order nonlinearities in the case of large ratchetting exponents, which can lead to numeral instabilities in finite-element implementations [32]. Hence, a ratchetting coefficient in the dynamic recovery term was introduced in [35]. A constitutive model including a plastic memory surface resetting scheme from Ohno et al. [36,37] accounting for the strain range dependency of cyclic hardening is adopted in [38] to 316 stainless steel for strain-controlled constant, pre-loading and ratchetting tests with positive mean strains at a temperature of 600 °C. Uniform ratchetting exponents are chosen for the five backstresses [38]. An exponential ratchetting formulation is used in [39] modifying the nonlinear kinematic hardening law of the Ohno–Wang model for modeling stress-controlled uniaxial ratchetting tests on austenitic stainless steel named Z2CND18.12N. The ratchetting behavior at room temperature of the duplex stainless steel S32750 is reasonably described in [40] with two additional constants entering the nonlinear Armstrong–Frederick hardening law by an accumulated plastic strain increment for both stress- as well as strain-controlled loading conditions. Based on the constitutive model of Abdel-Karim and Ohno [35], strain-controlled ratchetting of X12CrMoWVNbN10-1-1 steel at 600 °C is modeled in [41] with an exponential evolution equation for the mean stress relaxation parameter depending on the maximum plastic strain and accumulated cyclic plastic strain, since the amount of mean stress relaxation in the ratchetting experiment was found to be significantly dependent on the maximum plastic strain. Multiaxial ratchetting of duplex stainless steel (S32750) is modeled in [42] and of CS 1018 and CS 1026 steels in [43]. Okorokov et al. [44] capture the multiaxial ratchetting behavior of S355J2 steel with the introduction of two additional backstress super surfaces and a Dirac delta approach describing the stress deviation in uniaxial tension–compression and non-proportional axial-torsion tests.

These works mentioned show that ratchetting is a relevant phenomenon and that plasticity models have been developed to describe it. However, the works refer to isothermal conditions only, while non-isothermal conditions are hardly considered in the context of ratchetting. Stress-controlled TMF conditions with ratchetting are considered for a single crystal nickel-base superalloy in [45,46]. Ratchetting is modeled there by microstructural changes (rafting) and damage rather than with a modified kinematic hardening law. Models for cyclic ratchetting plasticity in the context of strain-controlled TMF loadings as relevant for this work are considered in [21,25] for nickel-based superalloys applicable to turbine blades, namely Haynes 230 and MAR-M247, respectively, and in [20] for copper relevant for rocket engines. A possible reason why ratchetting is considered rather less in plasticity models for TMF assessment of components is addressed in the following section.

### 1.3. Material Properties and Experiments

A major challenge when using plasticity models for thermomechanical loading is the determination of the material properties. On the one hand, appropriate experimental data from cyclic isothermal tests performed at different test temperatures in the relevant temperature range and from cyclic thermomechanical testing are required, which is time-consuming and, thus, expensive. On the other hand, the plasticity models include a relatively large number of temperature-dependent material properties that need to be determined based on experimental data. The more phenomena the model addresses, the more material properties are relevant, and the more specific experimental data are required, which includes information on the considered phenomena. An Armstrong–Frederick kinematic hardening model with static recovery, cyclic kinematic hardening and a ratchetting term, as it is, e.g., used in this work, already contains six material properties. However, depending on the application and loading conditions, usually, two or three backstresses are superimposed for fully reversed loading. If ratchetting is considered in the Armstrong–Frederick-type hardening law, up to ten backstresses are used to describe hardening curves realistically [34] since the ratchetting term changes the exponential hardening curve of one backstress to an almost bilinear curve. A maximum of ten backstresses is applied in this work, which gives 60 hardening-related and temperature-dependent material properties to be determined. This might be a reason why ratchetting models were hardly considered for thermomechanical loading so far.

To obtain reasonable results with the plasticity models, reasonable temperature dependencies of the material properties should be specified [9]. This typically includes, e.g., an increase of the viscosity and a decrease in strength with increasing temperature. If the material properties are determined from isothermal experimental data for each test temperature, respectively, so that a linear interpolation between the determined values gives a multi-linear temperature dependency, a reasonable temperature dependency will not be obtained automatically. This is especially true if a high number of materials properties is involved, e.g., by using several backstresses. Since little information is often given in literature on how temperature-dependent material properties are found, the authors of this work claim that it is best practice to use a combined experience-based and optimization-based approach to set some material property values manually from experimental evidence and to fine-tune these and other values numerically using optimization methods. This claim is confirmed by, e.g., [23,47]. Instead of using a multi-linear temperature dependency, relatively simple temperature-dependent functions for the material properties can be assumed a priori and the corresponding parameters used in the functions can be determined from the data, e.g., [14,16,26]. However, an a priori assumption of the functional temperature-dependency might limit the plasticity model’s capability to appropriately describe the isothermal and thermomechanical tests if the assumption does not represent the dependency reasonably.

For a Chaboche-type plasticity model that describes rate dependency as well as isotropic and kinematic hardening, a loading history for uniaxial strain-controlled isothermal material tests was designed in [48] that includes different strain amplitudes, different strain rates and hold times. It was shown that objective material properties (i.e., different persons determine the same values for the material properties from the same data) could be obtained from these so-called complex low-cycle fatigue (CLCF) tests if the material shows the phenomena considered in the model. Hence, the material properties can be determined from one experiment per temperature. CLCF tests have formed the basis for determining the temperature-dependent material properties of Chaboche-type viscoplasticity models for the evaluation of thermomechanical fatigue, e.g., [17,19,22]. A loading history that uses different mean strains was proposed to determine ratchetting-related material properties in [20,25].

### 1.4. Aims and Structure of the Paper

It can be summarized that time- and temperature-dependent plasticity models exist that cover relevant phenomena of the material behavior occurring in high-temperature components. For the application of these plasticity models, a relatively high number of material properties must be determined based on experimental data. Experiments considering loading histories designed to activate relevant phenomena can reduce the experimental effort to generate the required data.

However, strain-controlled time- and temperature-dependent cyclic ratchetting plasticity of nickel-based superalloys is hardly investigated, and appropriate data and models for the nickel-based alloy IN100 are, to the authors’ knowledge, not available. Hence, this paper aims to

- Characterize the time- and temperature-dependent cyclic ratchetting plasticity of IN100 using experiments with special loading histories designed to activate distinct deformation phenomena and to generate the required data for the determination and validation of the material properties;
- Propose a strategy for the determination of the multitude of temperature-dependent material properties on the basis of the generated isothermal data;
- Validate the model and the determined material properties using non-isothermal data.

The paper is structured as follows: the time- and temperature-dependent cyclic ratchetting plasticity model considered in this work is presented in Section 2. The experiments on IN100, including strain-controlled isothermal cyclic loading in the temperature range between 300 °C to 1050 °C as well as strain-controlled thermomechanical loading in this temperature range with different phase angles between temperature and mechanical strain, are described in Section 3. Section 4 explains in detail the strategy for determining the temperature-dependent material properties, followed by experimental and modeling results for the isothermal tests and the model validation by means of the thermomechanical fatigue tests. The experimental and modeling results are discussed in Section 5 and summarized in Section 6.

## 2. Time- and Temperature-Dependent Cyclic Ratchetting Plasticity Model

A Chaboche-type viscoplasticity model with nonlinear isotropic and kinematic hardening, static recovery, cyclic kinematic hardening and softening, e.g., [4], and the Jiang ratchetting model [34] is combined in this work to describe time- and temperature-dependent cyclic ratchetting plasticity. The Jiang model that is able to describe a constant ratchetting rate is used since a stable ratchetting rate under TMF loading conditions was found in [46]. In the following, the basic equations of the combined model are presented and the corresponding material properties, which are determined in Section 4 from the experimental data, are introduced. Since uniaxial test data are considered in this work, the uniaxial model formulation is described for simplicity.

In the model, the stress σ is determined from

(1) $$\sigma =E\left(\varepsilon -\varepsilon^{\mathrm{th}}-\varepsilon^{\mathrm{vp}}\right),$$

where *E* is Young’s modulus depending on temperature *T* and ε, $\varepsilon^{\mathrm{vp}}$ and $\varepsilon^{\mathrm{th}}$ are the total, the viscoplastic, and the thermal strain, respectively.

The thermal strain is obtained by the thermal expansion coefficient $\alpha^{\mathrm{th}}$ with respect to the reference temperature $T_{\mathrm{ref}}$ from

(2) $$\varepsilon^{\mathrm{th}}=\alpha^{\mathrm{th}}\left(T-T_{\mathrm{ref}}\right)-\alpha_{0}^{\mathrm{th}}\left(T_{0}-T_{\mathrm{ref}}\right).$$

With $\alpha_{0}^{\mathrm{th}}$ being the thermal expansion coefficient with respect to the initial temperature $T_{0}$, zero thermal strain as initial condition is obtained if $T_{\mathrm{ref}}\neq T_{0}$. The viscoplastic strain rate ${\dot{\varepsilon }}^{\mathrm{vp}}$ is obtained from the flow rule

(3) $${\dot{\varepsilon }}^{\mathrm{vp}}=\dot{p}\frac{\sigma -\alpha }{\left|\sigma -\alpha \right|},$$

where α is the backstress that describes kinematic hardening of the material. $\left|\;\cdot \;\right|$ indicates the absolute value. The backstress is additively decomposed into k=1,…,N backstresses

(4) $$\alpha =\sum_{k=1}^{N}\alpha^{\left(k\right)}.$$

The evolution equation for the backstresses is specified later in this section.

The equivalent viscoplastic strain rate is defined by a power-law function of the overstress

(5) $$\dot{p}={\dot{p}}_{0}{\langle \frac{\left|\sigma -\alpha \right|-\left(R_{e}+R\right)}{K}\rangle }^{n}.$$

$\langle \;\cdot \;\rangle$ represent the Macauley brackets. The temperature-dependent material properties *K* and *n* are the viscous reference stress and the rate sensitivity exponent, respectively. They quantify the viscous properties of the material and, thus, time-dependent effects at high temperatures, such as stress relaxation, creep, and strain rate dependency. Typical values for *n* are in the range of 3≤n≤30 [5]. The reference equivalent plastic strain rate ${\dot{p}}_{0}$ is introduced to ensure consistent units. $R_{e}$ is the temperature-dependent initial yield stress, and *R* describes isotropic hardening. In this work, exponential isotropic hardening is assumed to depend on the accumulated plastic strain *p* according to

(6) $$R=Q_{\infty }\left(1-e^{-bp}\right),$$

where $Q_{\infty }$ is the temperature-dependent saturation value and the temperature-dependent material property *b* that controls the isotropic hardening rate. Static recovery of isotropic hardening [4] has been shown not to be relevant for IN100; therefore, it is not considered in this work.

The evolution law for backstresses from Equation (4) is based on the Armstrong–Frederick kinematic hardening law but extended by static recovery, cyclic hardening/softening, and a ratchetting term:

(7) $${\dot{\alpha }}^{\left(k\right)}=C^{\left(k\right)}{\dot{\varepsilon }}^{\mathrm{vp}}-\gamma^{\left(k\right)}W^{\left(k\right)}\phi^{\left(k\right)}\dot{p}\alpha^{\left(k\right)}-R^{\left(k\right)}\alpha^{\left(k\right)}+\frac{\partial C^{\left(k\right)}}{\partial T}\frac{1}{C^{\left(k\right)}}\dot{T}\alpha^{\left(k\right)}.$$

The first term describes linear kinematic hardening with the temperature-dependent initial hardening modulus $C^{\left(k\right)}$. The second term, including the material property $\gamma^{\left(k\right)}$, decreases the hardening rate and is therefore called the dynamic recovery term [5] enabling a nonlinear hardening curve. The function $\phi^{\left(k\right)}$ is introduced to account for cyclic kinematic hardening or softening of the material:

(8) $$\phi^{\left(k\right)}=\phi_{\infty }^{\left(k\right)}+\left(1-\phi_{\infty }^{\left(k\right)}\right)e^{-\omega^{\left(k\right)}p}.$$

The material property $\omega^{\left(k\right)}$ controls the transition rate at which $\phi^{\left(k\right)}$ evolves from an initial value of 1 to an asymptotic steady state value $\phi_{\infty }^{\left(k\right)}$, depending on the accumulated plastic strain. Both material properties, $\phi_{\infty }^{\left(k\right)}$ and $\omega^{\left(k\right)}$, can depend on temperature.

Only the temperature dependency of the hardening modulus $C^{\left(k\right)}$ is accounted for in the temperature rate term [8], i.e., the last term in Equation (7). Thus, only for a constant (temperature-independent) value of $\gamma^{\left(k\right)}$, the backstress $\alpha^{\left(k\right)}$ is bounded for non-isothermal conditions by the current saturation value that accounts for the current state of cyclic hardening or softening:

(9) $$\alpha_{\infty }^{\left(k\right)}=\frac{C^{\left(k\right)}}{\phi^{\left(k\right)}\gamma^{\left(k\right)}}.$$

The fraction $C^{\left(k\right)}/\gamma^{\left(k\right)}$ is the isothermal saturation value of the Armstrong–Frederick kinematic hardening law without cyclic hardening and softening. Hence, $\phi_{\infty }>1$ results in cyclic kinematic softening and $\phi_{\infty }<1$ in cyclic kinematic hardening. Since the ratchetting function $W^{\left(k\right)}$ changes during the loading cycle in the range $0\leq W^{\left(k\right)}\leq 1$ (as defined in the following equation), it is not relevant for the saturation value. If, however, a temperature-dependent $\gamma^{\left(k\right)}$ is assumed, a temperature history-dependent behavior is obtained in which the backstress $\alpha^{\left(k\right)}$ is not bounded by its saturation value $\alpha_{\infty }^{\left(k\right)}$ anymore. This can result in unrealistic behavior under non-isothermal loading for an unfavorable temperature dependency of $\gamma^{\left(k\right)}$ [9].

Since the Armstrong–Frederick kinematic hardening law overestimates the ratchetting rate, the ratchetting function $W^{\left(k\right)}$ developed by Jiang [34] is introduced in the evolution Equation (7) for the backstress:

(10) $$W^{\left(k\right)}={\left(\frac{|\alpha^{\left(k\right)}|}{\alpha_{\infty }^{\left(k\right)}}\right)}^{\chi^{\left(k\right)}}.$$

The saturation value for the current state of cyclic hardening and softening is introduced in the combined Chaboche–Jiang model for scaling the backstress’s absolute value so that $0\leq W^{\left(k\right)}\leq 1$ is maintained. While Jiang defined functional dependencies for the ratchetting exponent $\chi^{\left(k\right)}$, it is used as a temperature-dependent material property in this work.

Finally, the third term in Equation (7) describes with the temperature-dependent material property $R^{\left(k\right)}$ static recovery of the backstress (i.e., a recovery with time), which is often observed at high temperatures. The used static recovery term results in an exponential decay of the backstress with time under isothermal conditions.

The time- and temperature-dependent cyclic ratchetting plasticity model presented in Equations (1)–(10) contains the following material properties that need to be determined dependent on temperature from experimental data:

- thermoelastic properties *E*, $\alpha^{\mathrm{th}}$ (Poisson’s ratio ν is not considered in this work),
- viscous properties *K* and *n* and
- plastic properties $R_{e}$, $Q_{\infty }$, *b*, $C^{\left(k\right)}$, $\gamma^{\left(k\right)}$, $\phi_{\infty }^{\left(k\right)}$, $\omega^{\left(k\right)}$, $\chi^{\left(k\right)}$ and $R^{\left(k\right)}$ with k=1,…,N.

In Section 4, up to N=10 backstresses (in accordance with [49]) are used to describe the experimental data. Hence, a total of 67 material properties are determined for each temperature used in the experiments. To determine the material properties, the plasticity model is implemented in its uniaxial formulation in a C++ environment.

## 3. Experiments and Set-Up

Section 3.1 briefly introduces the microstructure and chemical composition of the investigated nickel-based superalloy IN100 and the used specimen geometry. The testing procedure and loading histories for the experiments to identify the time-dependent deformation phenomena are described in Section 3.2.

### 3.1. Material and Specimens

**Figure 1:**
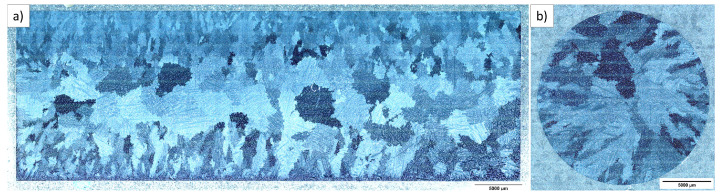
Metallographic sections of a cast bar under the light microscope in dark field mode in (**a**) crosswise and (**b**) lengthwise direction.

The tested material IN100 is a coarse-grained nickel-based superalloy. The blanks were produced as bars by vacuum investment casting from one single melt by ZOLLERN GmbH & Co. KG, Sigmaringendorf-Laucherthal, Germany. The blanks were then hot isostatically pressed at  1200 °C and 1030 bar for 4 h to minimize pores that occurred during the casting process. The bars have a diameter of  18 mm and a length of  120 mm. Metallographic sections of a blank are shown in Figure 1, and the chemical composition of the investigated material is given in Table 1. Grain sizes range from 250 μm to 1200 μm and increase from the surface of the bars to the center.

### 3.2. Testing Procedure and Data

Four types of experiments were carried out to determine the material properties of the presented plasticity model: CLCF, ratchetting, TMF and compression tests. The individual experiment types are described briefly in the following subsections.

**Figure A1:**
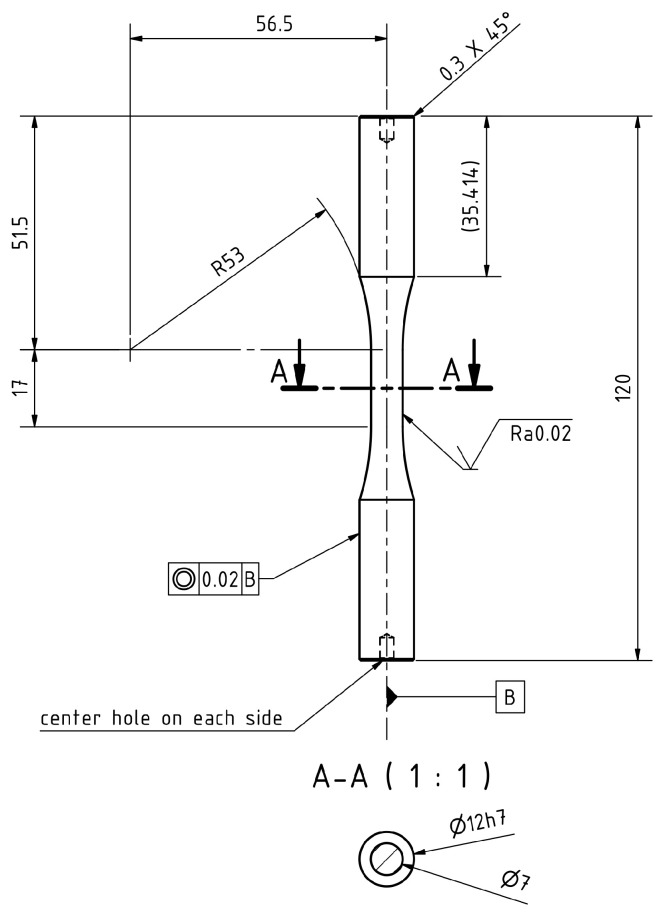
Technical drawing of the used specimens.

For the CLCF, ratchetting and TMF tests, cylindrical specimens with a parallel measuring range of  17 mm length were turned from the blank bars. Except for the clamping areas, the specimen surface was lapped to a roughness of $R_{a}=0.02$ μm. The specimen drawing is shown in the Appendix A in Figure A1. The tests were conducted with the use of an electromechanical testing machine of Walter + Bai with hydraulic clamping. Strain control was carried out by a Maytec high-temperature extensometer with a measuring range of  10 mm. The specimens were heated inductively, and temperature control was performed with a type K thermocouple in the center of the measuring range. Two additional thermocouples were placed at the lower and upper edges of the measuring range to monitor the temperature distribution throughout the measuring range. For the TMF tests, a nozzle for cooling the specimen with compressed air in the clamping section was additionally used.

The compression tests were performed in a Gleeble 3150 simulator from Dynamic Systems, Inc., where direct resistance is used for specimen heating. Small cylindrical specimens with a diameter of 5 mm and a respective length of 7.5 mm were manufactured from three blanks. The specimens are fixed in the Gleeble simulator between a water-cooled setup consisting of several layers of tungsten-carbide anvils. At the contact surfaces of the specimen and the anvils, thin graphite foils and a nickel lubricant are used to reduce friction and specimen bulging with increasing deformation. The temperature is measured with a type K thermocouple, which is welded in the middle of the specimen.

#### 3.2.1. Complex Low-Cycle Fatigue Tests

**Figure 2:**
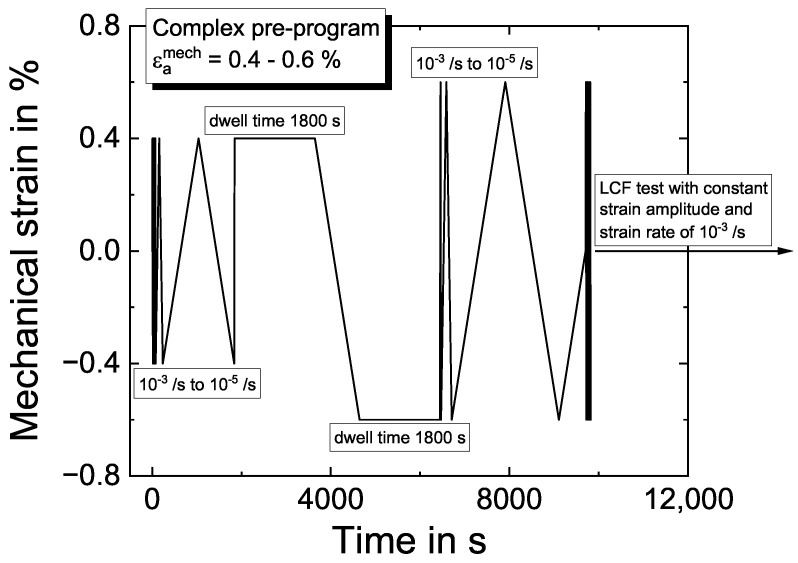
Strain-time history of the used pre-program for CLCF tests exemplary for a strain amplitude of 0.6%. The individual cycles are described in the respective subsection in detail.

The CLCF tests are composed of a complex pre-program shown in Figure 2, followed by a low-cycle fatigue (LCF) test, carried out according to [50] in a triangular cycle with a strain rate of $\dot{\varepsilon }=10^{-3}/s$.

The loading history of the complex pre-program includes three different strain rates of $\dot{\varepsilon }=10^{-5}/s,\;10^{-4}/s\;\mathrm{and}\;10^{-3}/s$ and dwell times of 1800 s in tension and compression with two different strain amplitudes, as shown in Figure 2. Three experiments were performed at temperatures of 300 °C, 750 °C, 850 °C, 950 °C and 1050 °C with the respective strain amplitudes of 0.15% to 0.2%, 0.2% to 0.3% and 0.4% to 0.6%. The values represent the strain amplitudes of the pre-program, where the load is increased between the two dwell times, and the second value also corresponds to the LCF test’s strain amplitude.

The pre-program starts with the lower strain amplitude and three triangular loading cycles with a strain rate of $10^{-3}$ /s, followed by each one loading cycle with 10^−4^/s and 10^−5^/s. A strain rate of $10^{-3}$ /s is chosen to enter the first 1800 s dwell time in tension. Between both dwell times, the strain rate is $10^{-5}$ /s, and the strain amplitude is increased, i.e., from 0.4% to 0.6%, as shown in Figure 2. After the second 1800 s dwell time, three loading cycles are applied with strain rates of $\dot{\varepsilon }=10^{-3}/s,\;10^{-4}/s\;\mathrm{and}\;10^{-5}/s$ until the fatigue test is continued with a typical LCF program. The structure of the pre-program was chosen to determine as many material properties of the used model as possible based on a few cycles. Results of CLCF tests are shown in Section 4.2.1, together with the modeling results.

#### 3.2.2. Ratchetting Tests

**Figure 3:**
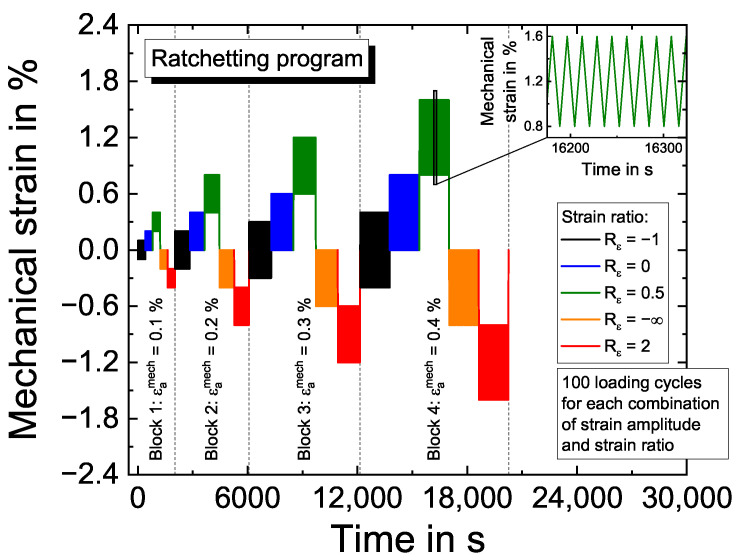
Strain-time history of the ratchetting program.

The strain-controlled ratchetting testing program is shown in Figure 3, which provides four loading blocks with strain amplitudes of 0.1%, 0.2%, 0.3% and 0.4% with each five strain ratios in order of $R_{\varepsilon }=-1$, 0, 0.5, −∞ and 2, respectively. Each combination of strain amplitude and strain ratio is tested for 100 loading cycles giving 500 cycles for each loading block. The range of the loading blocks is indicated by the vertical, gray dashed lines, while the different colors distinguish the strain ratios. The form of the loading cycles is triangular with a respective strain rate of $\dot{\varepsilon }=10^{-3}/s$, shown in the zoomed image window in the upper right corner of Figure 3. After the first loading block, the strain amplitude is increased to the next increment, and the strain ratios are varied in the order as specified. This procedure is repeated until specimen fracture occurs. The ratchetting tests are performed at temperatures of 300 °C, 750 °C, 850 °C, 950 °C and 1050 °C. Results of ratchetting tests are shown in Section 4.2.2, together with the modeling results.

#### 3.2.3. Compression Tests

Isothermal compression tests are performed to obtain the deformation behavior of the nickel-based superalloy over a large mechanical strain range since the CLCF, ratchetting, and TMF experiments include comparable small strain amplitudes. The results of the compression tests are used for adapting the viscoplasticity models to capture localized strain concentrations at cooling holes or channels or applying the plasticity models in fracture mechanics assessments of TMF cracks, respectively.

The compression tests were performed displacement-controlled in lab air with a respective strain rate of $\dot{\varepsilon }=10^{-3}/s$. The internal control software and a LabView interface provide data acquisition. In addition, a high-resolution sample image is captured every second during the test to determine the bulge diameter of the specimen. The logarithmic strain $\varepsilon_{\mathrm{true}}$ is calculated from the technical strain ε using the initial distance $l_{0}$ between the anvils and the reduction of distance Δl between the anvils during compression of the specimen with $\varepsilon_{\mathrm{true}}=\ln\left(1+\varepsilon \right)$. The true stress $\sigma_{\mathrm{true}}$ is obtained from the technical stress σ under the assumption of plastic incompressibility via $\sigma_{\mathrm{true}}=\sigma \left(1+\varepsilon \right)$. Friction and bulging effects are considered by taking the Siebel stress into account; see [51]:

(11) $$\sigma_{\mathrm{Siebel}}=\frac{\sigma_{\mathrm{true}}}{1+\left(\frac{2}{3}\frac{\mu_{f}d_{0}}{l_{0}}\exp\left(\frac{3}{2}p\right)\right)}.$$

A friction coefficient of $\mu_{f}=0.1$ is assumed [52]. In Equation (11), the quantity $d_{0}$ describes the specimen diameter, and *p* denotes the accumulated plastic strain with $p=\varepsilon_{\mathrm{true}}-\sigma_{\mathrm{true}}/E$, respectively, see [52].

Results of the compression tests are shown in Section 4.2.3, together with the modeling results. Since the investigated nickel-based superalloy exhibits large grain sizes of up to 1200 μm compared to relatively small specimens, the microstructure influences the resulting deformation and, thus, the measured stress–strain curves. Hence, three compression tests were performed for temperatures of 300 °C, 750 °C, 850 °C, 950 °C and 1050 °C, respectively. Due to the inhomogeneous grain size distribution, as displayed in Figure 1, premature shearing of the specimens occurred during experimentation at temperatures higher than 850 °C. The behavior is likely to be induced by slip along preferred slip planes in large grains in combination with the small specimen diameter, resulting in an inhomogeneous specimen deformation. This yields the measured stress to reach a stabilized plateau over an extensive strain range followed by a more or less pronounced decrease. For modeling, the stress–strain curve is extrapolated from the data where homogeneous deformations prevail to higher strains, as typically carried out for generating data for forming simulations with large strains, e.g., [53,54,55]. For the extrapolation, the Ramberg–Osgood [56] power-law hardening equation is used in this work. The Ramberg–Osgood equation is fitted to the experimental data in a strain range up to $\varepsilon_{\mathrm{true}}=5$% for each temperature. The data employed for the extrapolation are highlighted in Figure 9  in black, and the unused part of the compression curves in light gray, respectively. The Ramberg–Osgood extrapolation used for modeling is also shown in this figure. A slight scatter in strength between the three specimens is observed at 950 °C and 1050 °C. In the case of 1050 °C, the Ramberg–Osgood equation is adjusted only to the two experiments with higher strength levels.

#### 3.2.4. Thermomechanical Fatigue Tests

The TMF tests were performed under total strain control, according to [57]. The tests were performed in a temperature range of 300 °C to 950 °C and 300 °C to 1050 °C with the phase relationships in-phase (IP) and out-of-phase (OP). The heating and cooling rate is 5 K/s, and the total strain ratio is $R_{\varepsilon }=-1$, respectively. Results of the TMF tests are shown in Section 4.3, together with the modeling results.

## 4. Determination of Material Properties

In this section, the material properties of the time- and temperature-dependent cyclic ratchetting plasticity model introduced in Section 2 are determined based on the data of the experiments described in Section 3. To this end, different levels of complexity of the models are used to address the significance of the ratchetting term and the number of backstresses used:

- A pure Chaboche-type viscoplastic model without ratchetting term (i.e., $\chi^{\left(k\right)}=0$ in Equation (10)) with N=2 and N=5 backstresses in Equation (4);
- The fully combined Chaboche-type viscoplasticity model with Jiang ratchetting term with N=5 and N=10 backstresses in Equation (4).

The strategy for determining the multitude of material properties is presented in Section 4.1 before the experimental and modeling results are presented in Section 4.2. The model and the determined material properties are validated based on the TMF tests in Section 4.3 to account for a suitable temperature dependency of the material properties. All material properties of the viscoplasticity model are summarized in Appendix A in Table A1, Table A2, Table A3 and Table A4.

### 4.1. Strategy

In general, several approaches are possible to determine the values for the plastic and viscous material properties that give a good description of experimental data. The material properties can be determined experience-based, which means that a particular person assumes and decides on plausible material properties and their temperature dependency. The experience-based approach results in subjective material properties since the knowledge and experience of the person have a major influence on the results and, consequently, different persons would probably obtain other material properties for the same experimental data. Alternatively, optimization-based approaches, where an objective function (usually computed from a sum of least squares) is numerically optimized, can give objective material properties if the loading history used in the experiments activates all phenomena the plasticity model can describe and if the material itself shows the phenomena sufficiently [20]. Such objectivity, however, is hardly obtained for plasticity models with several backstresses. Moreover, it is hardly possible to obtain reasonable temperature dependencies for the material properties using an optimization-based approach. As the case for prescribed functions for the material property’s temperature dependency, the a priori introduction of constraints during optimization can limit the plasticity model’s capability to describe the experimental data appropriately.

This work uses a systematic experience-based approach to determine material properties with physically reasonable temperature dependencies. To this end, the experimental data of the isothermal tests are used. The experimental data are prepared for some material properties so that a subset of the data (e.g., only load reversal points) is employed to determine these properties. Other material properties are determined by calculating the complete data and by comparison with the experiment. The strategy and procedure to determine the material properties are explained in the following subsections.

#### 4.1.1. Thermoelastic Properties

The modulus of elasticity *E* is determined by linear regression with a strain tolerance of 10^−4^ from the loading and unloading loading branches from ten stabilized hysteresis loops at half lifetime from the CLCF tests for the different temperatures. The obtained values are similar for all plasticity models and given, e.g., in Table A1.

The coefficient of thermal expansion $\alpha^{\mathrm{th}}$ is determined from thermal strain curves measured in stabilized thermocycles during the zero stress test before starting the temperature–strain history in the TMF test with a temperature range of 300 °C to 1050 °C (see also Figure 10). Hence, $T_{\mathrm{ref}}=$300 °C. The corresponding values for the considered temperatures are also compiled, e.g., in Table A1.

#### 4.1.2. Viscous Properties

In the flow rule from Equation (3), the reference equivalent plastic strain rate ensuring consistent units is set to ${\dot{p}}_{0}=1/s$. For the material properties *K* and *n*, it is known that they show a relatively high correlation for strain rates relevant for components [48,58,59], i.e., different combinations of *K* and *n* result in almost the same response. This is why, in this work, the viscous exponent *n*, describing the stress sensitivity, is determined based on IN100 creep data documented in literature, assuming that the creep rate is proportional to $\sigma^{n}$. This is not consistent with the flow rule from Equation (3). However, the approach gives reasonable values for *n* from possible *K* and *n* pairs. With fixed *n*, the corresponding (unique) *K* is determined from the measured CLCF test data.

**Figure 4:**
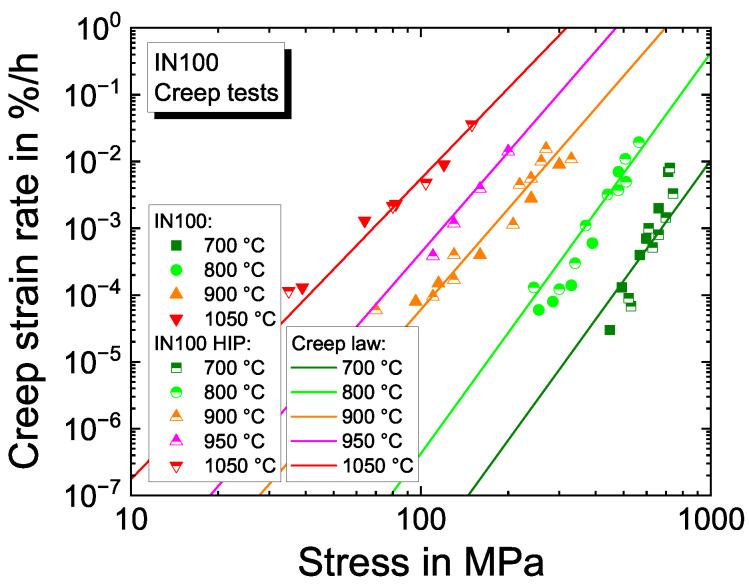
Experimental creep data from [60,61,62,63] and adjusted creep law from [64].

For the determination of *n*, experimental creep tests from previous national research projects [60,61,62,63] are analyzed; see Figure 4. The viscous exponent *n*, represented by the linear regression slope for the data of each temperature, decreases with increasing temperatures. The reference stress *K* is found with the given *n* iteratively with a starting value of  50 MPa and a monotonically increasing evolution with temperature, which fits the time-dependent material behavior in the complex pre-program of the CLCF tests with good accuracy.

#### 4.1.3. Plastic Properties: Isotropic Hardening

**Figure 5:**
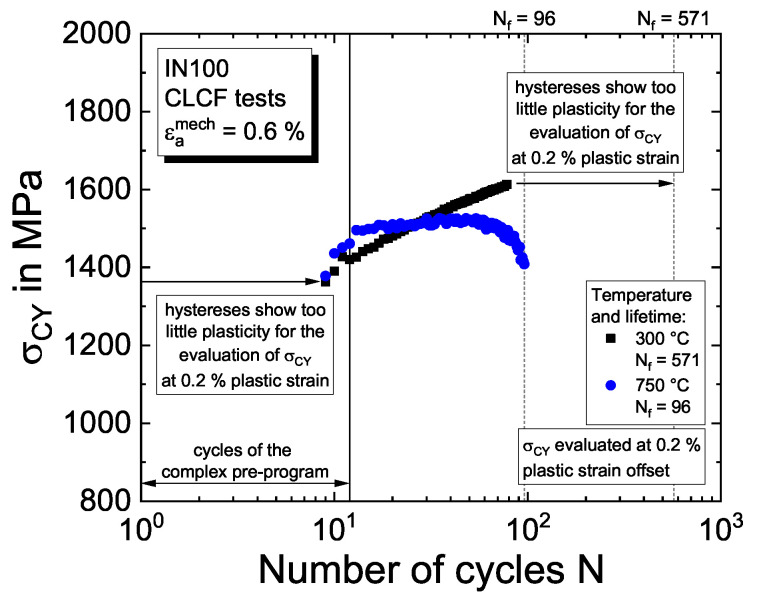
Evolution of the cyclic yield stress $\sigma_{\mathrm{CY}}$ for 0.2% plastic strain offset and temperatures of 300 °C and 750 °C.

For each loading cycle of the CLCF tests for each temperature, the cyclic yield stress with respect to the point of load reversal is determined using a plastic strain offset of  0.2%. The results are shown in Figure 5 for temperatures of 300 °C and 750 °C, for which an evolution of the cyclic yield stress is observed. The lifetime $N_{f}$ determined by a five percent drop in the maximum stress is denoted by the vertical dashed lines. From the evolution of the half cyclic yield stress as a function of the accumulated plastic strain in the experiment, which is computed from the summation of the doubled plastic strain range of each cycle, the initial yield stress $R_{e}$ and the isotropic hardening properties in Equation (6) are determined by fitting the exponential function.

#### 4.1.4. Plastic Properties: Kinematic Hardening

The most time-consuming task is the determination of the material properties related to kinematic hardening (Equation (7)). The strategy to determine these properties in this work is related to the one described in [49].

First, the number of backstresses *N* is chosen in a range, so that the experimental hysteresis loops are reproduced with close accuracy and a smooth shape. This choice is subjective. Considering the increase in the number of material properties and the computation time for the model with the increase in the number of backstresses, the number of backstresses should be kept low. In [49], a plasticity model is presented for 1070 steel, material properties are given for 5 as well as for 10 backstresses, and it is recommended to use N=5…10 backstresses. For 10 backstresses, the experimental data are described with higher accuracy, and the hardening curves in the stress–strain hysteresis loops show a smoother shape. For the model with 5 backstresses, the predicted stress–strain hysteresis loop exhibits bending points at the transition between the individual backstresses.

In this work, 5 and 10 backstresses are chosen for modeling the behavior of IN100 nickel-based superalloy with the ratchetting term. A number of 2 and 5 backstresses are used for the formulation without ratchetting. In all cases, starting values for the saturation constants $C^{\left(k\right)}/\gamma^{\left(k\right)}$ and the dynamic recovery constants $\gamma^{\left(k\right)}$ are determined as illustrated in [49]. In this reference, the experimental $\Delta \sigma -\Delta \varepsilon^{\mathrm{pl}}$ curve is approximated piecewise linear by the additive decomposition of the backstress; see Equation (4). To avoid undesirable temperature history effects in the case of non-isothermal loading histories, temperature-independent recovery constants $\gamma^{\left(k\right)}$ are used in this work. Moreover, a monotonic decrease of $\gamma^{\left(k\right)}$ for increasing superscripts k=1…10 is reasonable. Here, backstress k=1 describes the regions of the hardening curve with higher hardening rates for smaller plastic strains. Backstress k=10 finally covers the region with a lower hardening rate for the highest plastic strain.

A complete saturation of all backstresses at high plastic strains can lead to instability and convergence problems in finite-element calculations due to the then-present perfectly-plastic behavior. The largest strain amplitude in CLCF experiments is $\varepsilon_{a}^{\mathrm{mech}}=0.6$%. To ensure hardening at higher plastic deformations, as can occur at points with strain localization in components, in crack tip fields or during the iterative solution in a finite-element calculation, the deformation behavior is extrapolated to large-strain loading amplitudes from the compression tests.

A large incrementation between the first and last recovery constant $\gamma^{\left(k\right)}$ ensures that the model adequately describes a large loading range with >50% plastic strain amplitude. The constants $C^{\left(k\right)}/\gamma^{\left(k\right)}$ are fitted with monotonically decreasing saturation values for ascending superscripts *k* and increasing temperatures *T*. Only the saturation value of the last backstress (k=10) is slightly increased to provide reasonable strain hardening for huge deformations.

**Figure 6:**
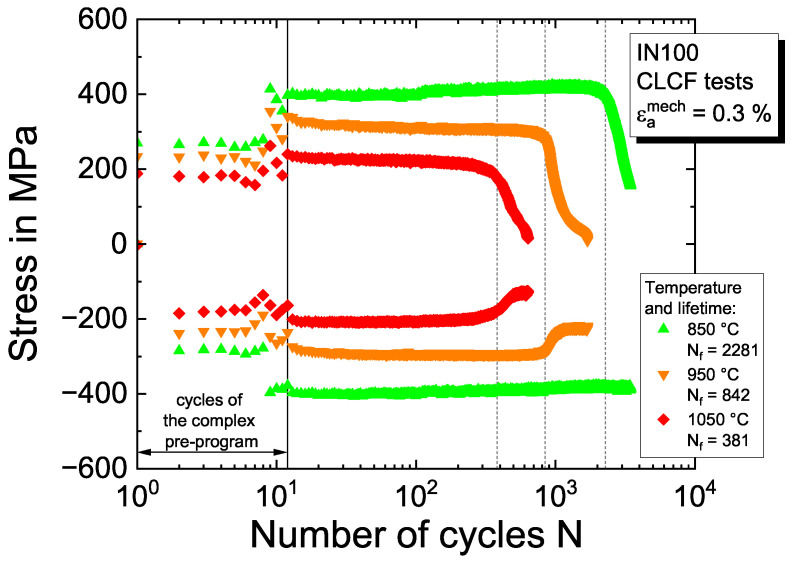
Evolution of the stress at the load reversals of the cycles in the CLCF tests with a strain amplitude of 0.3% and temperatures of 850 °C, 950 °C and 1050 °C.

The material properties of the function $\phi^{\left(k\right)}$ for the description of cyclic kinematic hardening and softening, i.e., the saturation values $\phi_{\infty }^{\left(k\right)}$ and the progression constants $\omega^{\left(k\right)}$, are determined from the evolution of the stress at the load reversals of the cycles. As the material shows isotropic cyclic hardening at temperatures of 300 °C and 750 °C and a stabilized cyclic material behavior for the intermediate temperature of  850 °C, the saturation values $\phi_{\infty }^{\left(k\right)}=1$ are used for these temperatures (keeping $\phi^{\left(k\right)}=1$) and $\omega^{\left(k\right)}=0$. As indicated by the evolution of the stress at the load reversals of the cycles in Figure 6 for the intermediate strain amplitude of $\varepsilon_{a}^{\mathrm{mech}}=0.3$%, temperatures above  850 °C lead to increasing cyclic softening so that $\phi_{\infty }^{\left(k\right)}>1$. The rate of softening is controlled by the transition constants $\omega^{\left(k\right)}$ and fitted to the evolution of the stress at the load reversal points after the complex pre-program. For reasons of simplicity, the values for both properties are chosen to be equal for all backstresses.

The material properties $R^{\left(k\right)}$ related to the static recovery of the backstresses are found by considering the amount of stress relaxation during the dwell times of the complex pre-program. Since no information about the individual contribution of the respective backstress on the experimentally observed stress relaxation is obtained from the experimental data, identical $R^{\left(k\right)}$ values are assumed for each backstress. The stress relaxation phenomenon is accompanied by increasing temperatures and, thus, $R^{\left(k\right)}$ is also monotonically increased. An exponential dependency would be also reasonable since thermally activated processes drive recovery of hardening. A linear dependency, however, gives a good description of stress relaxation.

Finally, the ratchetting exponents $\chi^{\left(k\right)}$ are adjusted based on the ratchetting experiments. In accordance with [49], the values of the ratchetting exponents $\chi^{\left(k\right)}$ are selected in ascending order with an increasing number of backstress k=1…10. Ratchetting exponents with small superscripts *k* mainly control the initial ratchetting rate after changing the strain ratio, while the high superscript exponents $\chi^{\left(k\right)}$ determine the long-term ratchetting rate; see [49]. Due to [49], no ratchetting is predicted for $\chi^{\left(k\right)}\to \infty$. Since the mean stress relaxation becomes more pronounced with increasing temperature, the ratchetting exponents are consequently decreased.

### 4.2. Results for Isothermal Experiments

The material properties determined from the isothermal tests according to the strategy described in the previous section are compiled in the Appendix A in Table A1, Table A2, Table A3 and Table A4. The experimental and model results are compared in the following subsections for the isothermal tests.

#### 4.2.1. Complex Low-Cycle Fatigue Tests

**Figure 7:**
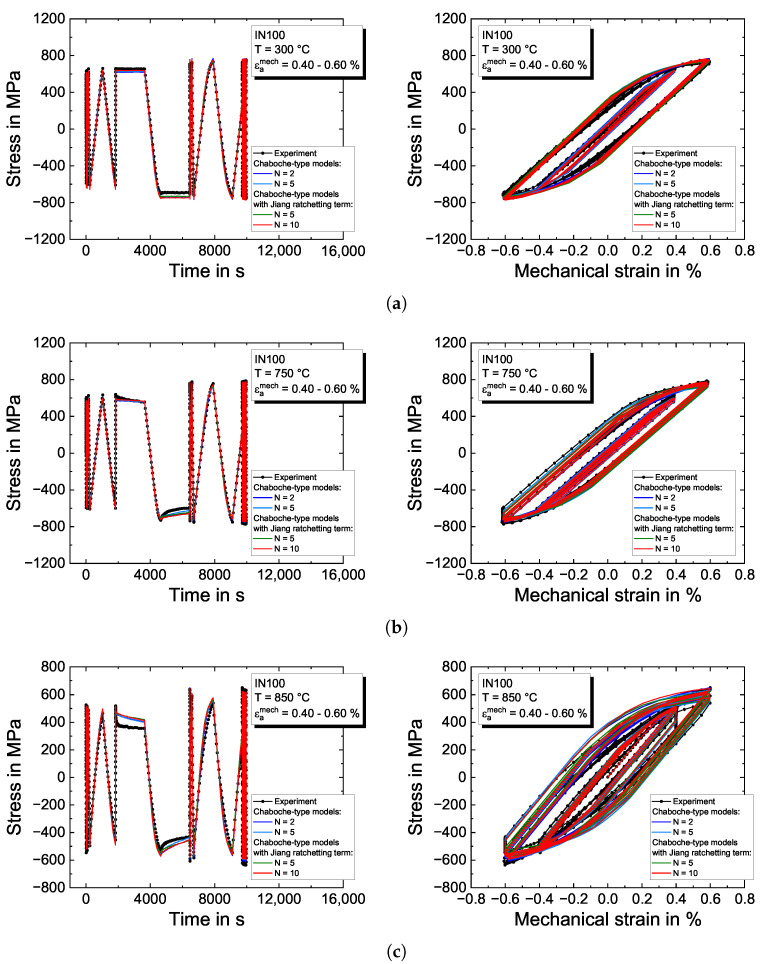

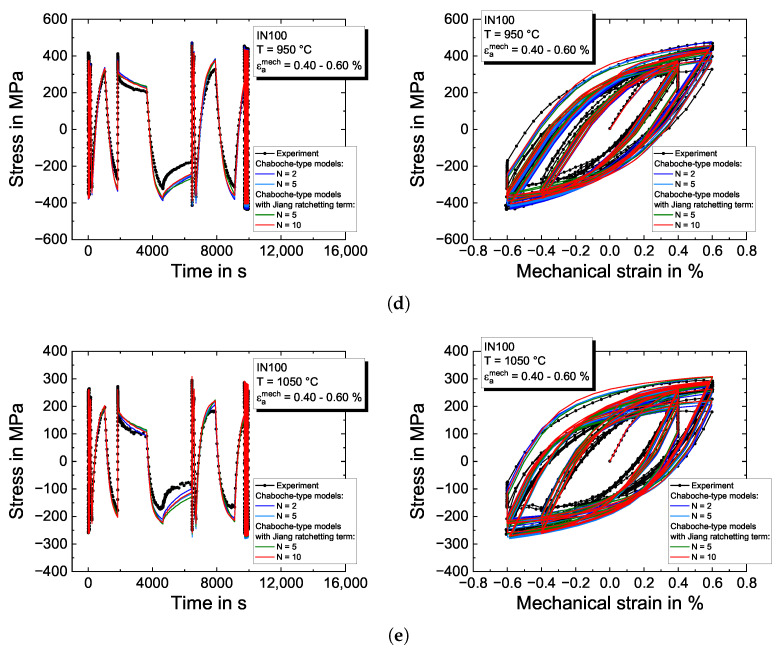
Modeling results of the Chaboche-type viscoplasticity models for the pre-program of the CLCF tests on the nickel-based superalloy IN100 for (**a**) 300 °C; (**b**) 750 °C; (**c**) 850 °C; (**d**) 950 °C; and (**e**) 1050 °C. The left figures show the stress–time evolution and the right figures the stress–strain hysteresis loops, respectively.

The experimental results of the isothermal complex pre-program from CLCF tests and results of the plasticity models with the determined material properties are presented in Figure 7 for a mechanical strain amplitude of 0.4% to 0.6%. The dotted black lines characterize the experimental results, while the model responses are plotted in colored lines. The stress–time evolution is shown in the left column and the corresponding stress–strain hysteresis loops in the right column.

On the left side in Figure 7a for 300 °C, the material shows slightly cyclic hardening effects in the first loading cycles. A stationary stress level is observed in the dwell times. The increase of the initial mechanical strain amplitude of $\varepsilon_{a}^{\mathrm{mech}}=0.4$% up to $\varepsilon_{a}^{\mathrm{mech}}=0.6$% in the unloading cycle between both dwell times results in only marginally higher stresses of around  750 MPa. The model slightly overestimates the compressive stress level during the second dwell time. Strain rate-dependent effects are not observed at this temperature. In the LCF part, cyclic hardening of the material is well described by the viscoplasticity models by activating the isotropic hardening material properties. The deformation behavior is shown on the right side of Figure 7a by means of the stress–strain hysteresis loops. The model predicts the elastic stiffness somewhat higher compared to the experimental results. The plasticity models can describe the curves adequately and ensure smooth transitions between the individual backstress evolutions.

Increasing the temperature to 750 °C and 850 °C leads to a reduction in the overall material strength and to increasing stress relaxation in dwell times, shown in Figure 7b,c. Furthermore, strain rate dependency is observed at a temperature of 850 °C. The time-dependent effects are reasonably described with the determined viscous properties *K*, *n* and the material properties for the static recovery $R^{\left(k\right)}$. However, there is some deviation after the first dwell time where the model overestimates the strength during compressive loading before the second hold time. In the LCF after the complex pre-program, the material exhibits at 750 °C slight cyclic isotropic hardening described by Equation (6); see Figure 7b. The viscoplasticity models almost perfectly describe the corresponding stress–strain curves. However, the hysteresis loops in Figure 7c for the temperature of 850 °C show a slight deviation from the experimental stress level around zero strain. In contrast, the resulting minimum and maximum stresses are well covered by the model.

The experimental temperatures of 950 °C and 1050 °C result in a further reduced material strength and lead to a pronounced time-dependent material behavior in the complex pre-program presented in Figure 7d for 950 °C and Figure 7e for 1050 °C, respectively. The introduction of dwell times leads to remarkably fast stress relaxation at the beginning of the dwell time. The stress relaxation phenomenon is retarded; however, the stresses are not completely stabilized at the end of the dwell time. As the strain rate is varied, the hardening behavior is drastically reduced and shows almost perfectly-plastic deformation behavior at high mechanical strains and slow strain rates, which is especially displayed in the stress–strain hysteresis loops in Figure 7e for 1050 °C. For both temperatures, cyclic softening in the LCF part of the tests is captured by the function $\phi^{\left(k\right)}$ from Equation (8). Generally, all models give a visually good description of the CLCF tests.

#### 4.2.2. Ratchetting Tests

**Figure 8:**
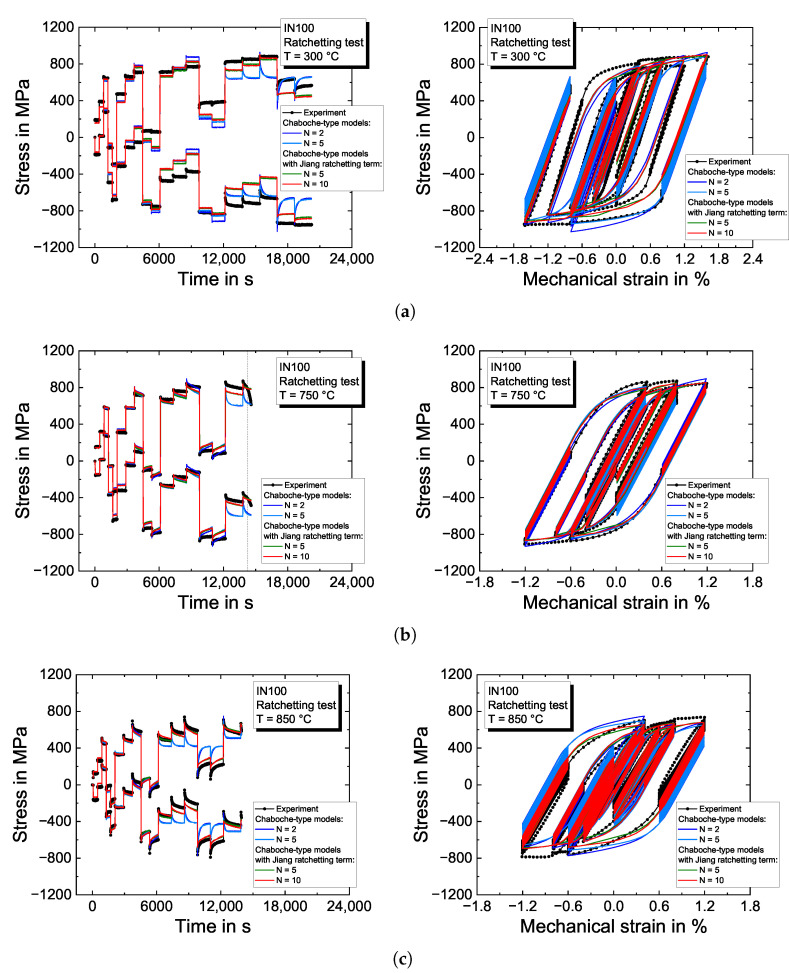

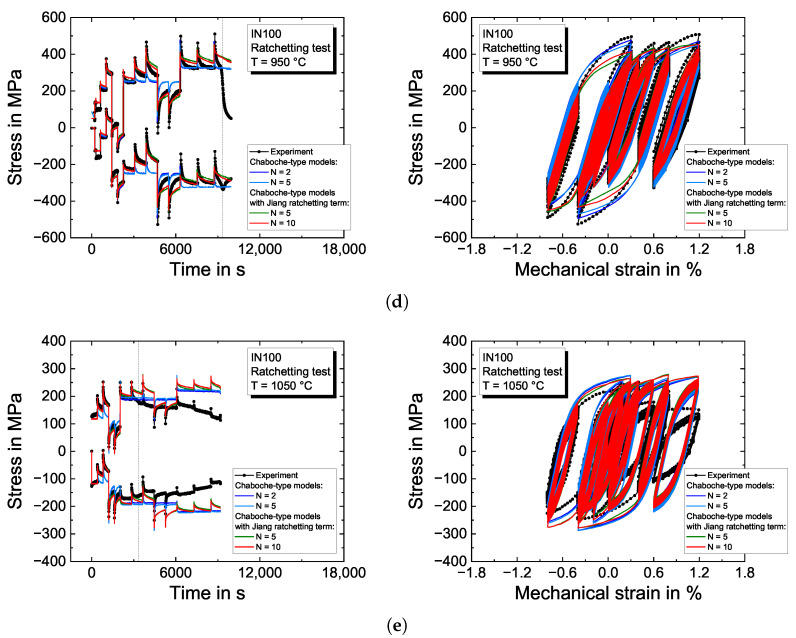
Modeling results of the Chaboche-type viscoplasticity models for the ratchetting experiments on the nickel-based superalloy IN100 for (**a**) 300 °C; (**b**) 750 °C; (**c**) 850 °C; (**d**) 950 °C; and (**e**) 1050 °C. The left figures show the evolution of the stress at the load reversal points and the right figures the stress–strain hysteresis loops, respectively.

The model predictions for the ratchetting tests for the five investigated temperatures are presented in Figure 8, together with the experimental results. At  300 °C in Figure 8a, no ratchetting effects are observed at the beginning of the experiment. However, as the strain amplitude is increased to $\varepsilon_{a}^{\mathrm{mech}}=0.3$%, the experimentally obtained stresses in tension are described appropriately, while the compression stress level is underestimated by ≈200 MPa. The Chaboche-type viscoplasticity models without ratchetting term (blue curves) cannot describe the experimentally observed stress evolution after a change in the strain ratio and predict a fast decrease of the mean stress.

For both intermediate temperatures of 750 °C and 850 °C in Figure 8b,c, the ratchetting effects in the experiments are predicted adequately by the viscoplasticity models with ratchetting term. Ongoing with higher testing temperatures, the material strength is reduced, while the decay in stress is increased significantly when the strain ratio is varied. Figure 8d illustrates a good correlation between the experiment and models for a temperature of  950 °C if ratchetting is considered in the viscoplasticity model. Without ratchetting, the ratchetting rate is overestimated by the model. Due to the high-temperature level, the specimen’s lifetime is reduced in the test, which is indicated by the stress drop at the end of the experiment and the vertical line at a decline of  5% in maximum stress. At  1050 °C in Figure 8e, the model almost perfectly describes the experimentally obtained stress evolution at the load reversal points. However, crack initiation is further accelerated and occurs at the very beginning of the ratchetting test in the second loading block resulting in a continuous strength decrease. Since the viscoplasticity models do not account for fatigue crack initiation and subsequent crack growth, the stresses are overestimated from the point of crack initiation, indicated by the vertical line. Nevertheless, the stress decay is quantitatively well reproduced.

#### 4.2.3. Compression Tests

**Figure 9:**
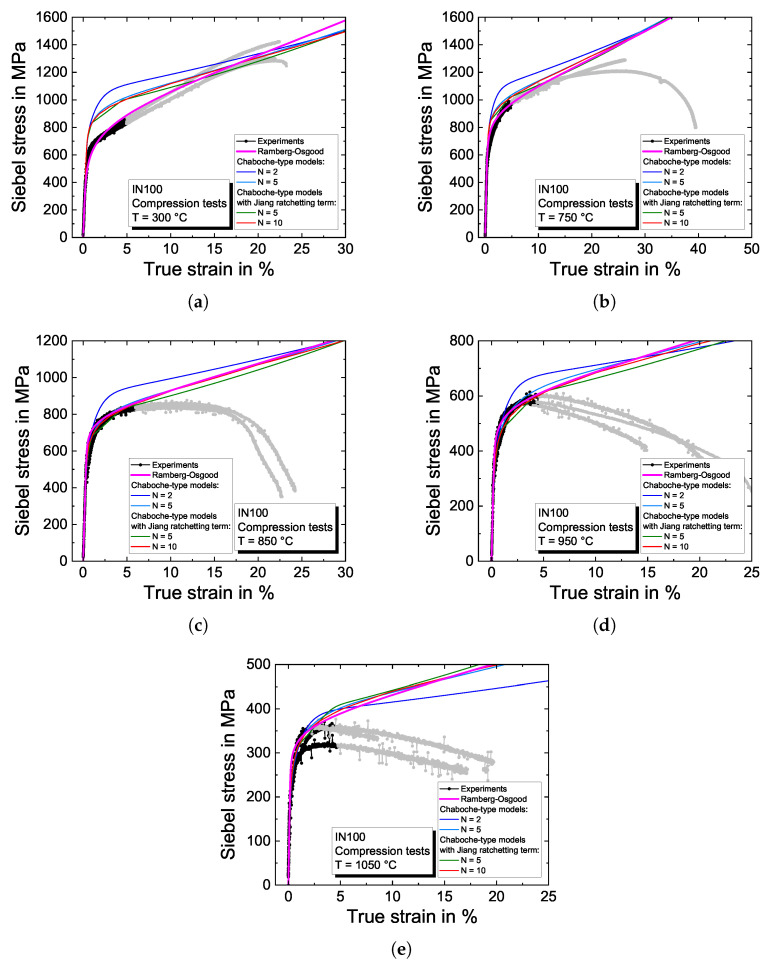
Modeling results of the Chaboche-type viscoplasticity models for the compression tests on the nickel-based superalloy IN100 for (**a**) 300 °C; (**b**) 750 °C; (**c**) 850 °C; (**d**) 950 °C; and (**e**) 1050 °C.

Figure 9 shows the results of the compression tests and the model predictions. The Siebel stress calculated according to Equation (11) is plotted against the true strain. Absolute values are displayed. The material properties are determined based on the extrapolated stress–strain curves obtained from the Ramberg–Osgood [56] equation.

The compression tests at a temperature of 300 °C in Figure 9a show almost linear hardening. The deformation behavior observed in the compression tests is not adequately described by the Chaboche-type viscoplasticity models, which were predominantly adapted to the cyclic experiments showing a significantly higher yield stress than the compression tests, where the point of yielding occurs at around 600 MPa. The overestimation is also addressed to isotropic hardening since, in the cyclic experiments, only a little plastic strain is accumulated even for the highest applied strain amplitude of 0.6%. The backstresses are adjusted to describe the experimental data at specimen failure reasonably. For the higher temperatures, the plasticity models, except the Chaboche-type model with N=2 backstresses, can describe the extrapolated stress–strain curves in Figure 9 with the determined material properties quite suitable. With N=2 backstresses, there is not enough flexibility in the model to describe hardening for smaller and higher strains simultaneously.

### 4.3. Model Validation on Thermomechanical Fatigue Tests

**Figure 10:**
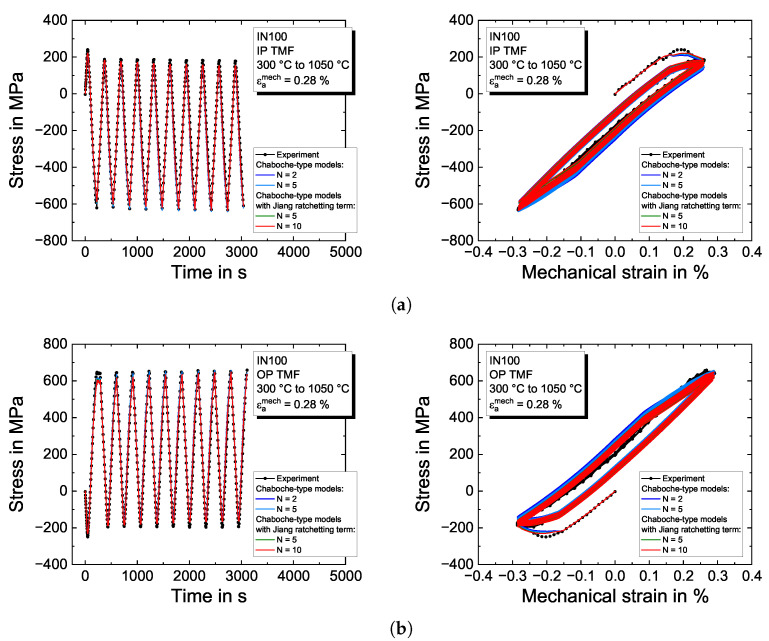
Modeling results of the Chaboche-type viscoplasticity models for the TMF experiments on the nickel-based superalloy IN100 for (**a**) IP TMF and (**b**) OP TMF between 300 °C to 1050 °C and a mechanical strain amplitude of $\varepsilon_{a}^{\mathrm{mech}}=0.28$%. The left figures show the stress–time evolution and the right figures the stress–strain hysteresis loops, respectively.

The temperature dependence of determined material properties and the ability of the plasticity models to describe non-isothermal conditions (even though the material properties were determined from isothermal tests) are validated with the TMF tests. A linear interpolation of the material properties in temperature is applied. The model predictions and the experimental results are shown in Figure 10 for IP and OP TMF loading with a temperature range between 300 °C and 1050 °C and a mechanical strain amplitude of $\varepsilon_{a}^{\mathrm{mech}}=0.28$%. IP TMF loading in Figure 10a results in a negative mean stress evolution. The maximum tensile stresses are observed in the initial loading cycle, but from the second loading cycle on, stabilized stress–strain hysteresis loops are obtained. For the applied mechanical strain amplitude, only little plasticity is observed. The model is capable of describing the experimentally observed curve very well. This is also the case for the corresponding OP TMF test in Figure 10b. In contrast to IP TMF loading, a positive mean stress evolution is adjusted. In addition, the larger opening of the hysteresis loops indicates higher amounts of plasticity than the IP TMF test.

**Figure 11:**
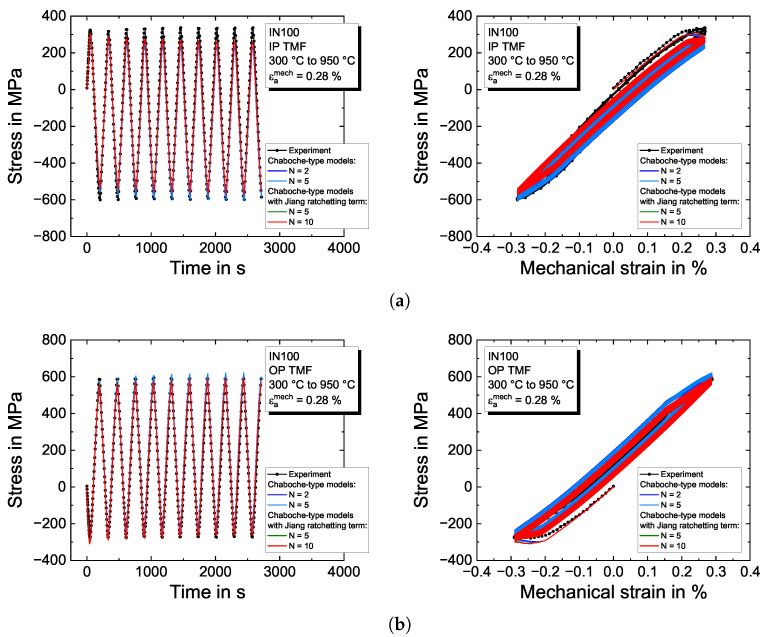
Modeling results of the Chaboche-type viscoplasticity models for the TMF experiments on the nickel-based superalloy IN100 for (**a**) IP TMF and (**b**) OP TMF between 300 °C to 950 °C and a mechanical strain amplitude of $\varepsilon_{a}^{\mathrm{mech}}=0.28$%. The left figures show the stress–time evolution and the right figures the stress–strain hysteresis loops, respectively.

Furthermore, the TMF tests with a temperature range between 300 °C and 950 °C and a mechanical strain amplitude of $\varepsilon_{a}^{\mathrm{mech}}=0.28$% are predicted by the viscoplasticity models in Figure 11. The results of the IP TMF test are presented in Figure 11a. The sample exhibits a higher stiffness than predicted by the models, which is attributed to the coarse-grained microstructure of the nickel-based cast IN100 alloy and leads to a scatter in Young’s modulus *E*. Accompanied by the lower maximum temperature of  950 °C, higher stresses in tension are achieved. The model slightly underestimates the maximum stresses in tension as well as in compression. However, the models almost perfectly describe the OP TMF test in Figure 11b. Generally, all models describe the TMF tests very well, even if only two backstresses are used for the pure Chaboche-type model.

In general, it is noted that the TMF tests show relatively little plasticity, which facilitates the description of the tests by the viscoplasticity models. No TMF tests were performed with any mean strain (e.g., $R_{\varepsilon }=0$ in the case of IP and $R_{\varepsilon }=-\infty$ in the case of OP). Hence, if the presence of a mean strain also leads to mean stress, which only the two plasticity models with ratchetting functionality can describe, remains an open question.

## 5. Discussion

In this work, the time- and temperature-dependent cyclic ratchetting plasticity of the nickel-based alloy IN100 is investigated in experiments with loading histories activating phenomena related to the material’s time-dependent as well as hardening and ratchetting behavior under strain control. Based on the data, the temperature-dependent material properties of a Chaboche-type time-dependent plasticity model, including the Jiang ratchetting term, are determined using an experience-based approach. Four different levels of complexity were considered: the pure Chaboche-type model without ratchetting term with N=2 and N=5 backstresses and the combined Chaboche–Jiang model with N=5 and N=10 backstresses.

In the uniaxial experiments, strain amplitudes, strain rates, mean strains and dwell times are chosen such that they are expected to be relevant for the assessment of stresses and strains as well as for fracture mechanics assessment of TMF cracks at cooling holes and channels in IN100 turbine blades and nozzle guide vanes. All experiments were run under strain control representing the TMF situation of constrained thermal strains where local plasticity is obtained. Stress-controlled conditions are not considered in this work since the stresses induced by centrifugal forces in rotating machinery are typically found below the yield stress. Nevertheless, the local stresses in components are the result of both strain- and stress-controlled loadings. Such a combination is, however, not possible in uniaxial experiments where either strain or stress control can be applied. Indeed, multiaxial tests such as tension–torsion tests or tests with biaxially-loaded cruciform specimens could be run with mixed control. However, the effort to determine the material properties from such tests increases significantly, and the effort for the determination of the material properties on the basis of uniaxial data are already relatively high. Moreover, the experiments in this work use uniaxial loading only so that the defined loading histories can be applied in material tests with standard TMF testing systems available at many testing facilities.

The investigated IN100 has relatively large grains compared to the dimensions of the specimen. This results in variations in the mechanical response of different specimens where local grain orientations can result in anisotropic behavior and control the average stress measured in the strain-controlled tests. Such effects are not considered in this work since a phenomenological macroscopic modeling approach for the finite-element assessment is used. It is assumed that the material properties determined for a specific temperature based on several tests with different specimens at this temperature describe an average behavior for the coarse-grained IN100. This assumption is supported by the observation that the behavior of the non-isothermally tested specimens can be well described on the basis of the material properties determined from the isothermally tested specimens. The variations induced by the coarse-grained microstructure could be assessed with single-crystal plasticity finite-element analysis of the specimen. However, this goes beyond the focus of this work.

In the following, the results obtained from the four models with different levels of complexity are discussed based on a visual assessment. This is justified since a combined experience- and optimization-based approach is applied when determining the material properties. This approach is, per se, non-objective and includes visual assessments of the quality of the model description. An objective approach would require a quality measure, e.g., a least square functional.

All models can give a good visual description of the CLCF tests with the material properties determined by the proposed strategy, i.e., the models can reproduce the decreasing stress levels and the increasing time dependency with increasing temperature. Furthermore, cyclic hardening and the behavior for higher strains from the extrapolated data are described well. The models also give a very good description of the TMF data, being the most relevant regarding the finite-element assessment of thermomechanically-loaded components. Hence, all models and the respective material properties could be validated for non-isothermal loading. The pure Chaboche-type models provide an equally good description, although the number of material properties is lower with only two backstresses. As can be expected when having no ratchetting term in the Armstrong–Frederick kinematic hardening law, mean stress relaxation is significantly overestimated by the pure Chaboche-type models for the experiments with mean stresses. By calibrating the ratchetting exponents, mean stress relaxation is decelerated so that the combined Chaboche–Jiang models give a good description of the mean stress relaxation of IN100. Indeed, the model response for the tests with mean strain shows some deviations compared to the experimental data. Such loading conditions with varying amplitudes might need some strain range memory effect to be accounted for in the model, e.g., as the extension of the Armstrong–Frederick hardening law in [29]. This effect might act superimposed on ratchetting, so that the effects cannot be separated in the data. Additional tests without mean strain but varying strain amplitudes would be necessary to have further information to evaluate memory effects.

When using N=5 backstresses, the stress–strain hysteresis loops show slight bending points due to the transition where one backstress comes to saturation, and another backstress becomes dominant. When using N=10 backstresses, the kinks are smeared out, and smooth curves are obtained. The efforts for modeling ratchetting with the multitude of material properties are high. From an application point of view, the plasticity models should offer added value in finite-element calculations to evaluate the behavior and lifetime of components. Thus, the question arises as to what extent the models with different complexity levels affect the results of finite-element analyses of components. If a significant sensitivity of ratchetting on the component level is given, either qualitatively or quantitatively, the materials testing and modeling efforts could be justified. This aspect will be taken up again in the following.

For the determination of the material properties, a step-by-step procedure based on subsets of experimental data is used. Moreover, some "rules" are defined regarding temperature dependency as, e.g., temperature-independent $\gamma^{\left(k\right)}$ to avoid temperature-history effects and unrealistic behavior as described in [9] as well as, e.g., increasing *K* and decreasing $C^{\left(k\right)}/\gamma^{\left(k\right)}$ with increasing temperature. Moreover, the same $R^{\left(k\right)}$ values for static recovery are used for all backstresses, and the ratchetting exponent $\chi^{\left(k\right)}$ increases as *k* increases. These rules provide a good overall description of the experimental data. Nevertheless, the material properties are non-objective since values are chosen based on experience and visual assessment of the data. Objectivity becomes increasingly important since material properties are used in material databases, which allow for the comparison of different materials and the analysis regarding, e.g., process–structure–property correlations and chemical composition. With the step-by-step procedure and the rules described and used in this work, different persons will still obtain different material properties. Analytical methods exist to assess the stability and the robustness of determined material properties, e.g., using eigenvalues of the Hessian matrix of a least square functional [20,48,65] or the covariance matrix [20,48,66].

However, these methods rely on “optimal solutions” where the deviation between the model response and the experimental data are vanishing small, and the solution is defined by a (local) minimum of some cost function. This is not the case here. The analytical methods are typically used with synthetically generated data with no model error to evaluate specific loading histories for their suitability for determining material properties [20,48,65]. It would be useful to perform a numerical assessment of the stability and robustness by using exclusively numerical optimization methods to determine the material properties starting from different initial guesses or by giving the task of determining the material properties to several people. From the quantitative analysis of the scatter in the determined material properties, the stability and robustness can be evaluated. Such analysis is planned for future works, which requires stronger restrictions concerning the temperature dependency of the material properties so that objectivity can be possible. Hence, the rules defined in this work can be taken as a basis for generalizing temperature dependencies and defining an optimization-based workflow for determining material properties that also apply to other nickel-based alloys.

Here, again, the question arises as to what extent the lack of objectivity of the material properties has an effect on finite-element calculations of components. Assume that different persons perform the same finite-element calculations, however, using other (non-objective) material properties giving all a visually good description of the same underlying experimental data. If these persons obtain considerably different results in the finite-element calculations, the results lose their significance.

The material data, material properties, and a user material subroutine UMAT for the finite-element program ABAQUS implementing the three-dimensional formulation of the combined Chaboche–Jiang plasticity model are available on request from the authors of this work so that collective experience can be gained in determining objective or non-objective material properties and their influence in finite-element calculations with components.

## 6. Conclusions

The paper comprises an efficient material characterization for the nickel-based superalloy IN100 for the application of time- and temperature-dependent cyclic ratchetting plasticity models. The results are concluded as follows:

- Experiments are used to activate time-dependent deformation phenomena in low-cycle fatigue tests with a preceding complex strain–time pre-program while the mean stress behavior for mean strain loading histories is investigated in ratchetting tests. Additionally, compression tests are performed to obtain insights into the deformation behavior over a large strain range;
- A step-by-step experience-based approach is presented to adjust Chaboche-type viscoplasticity models with four different levels of complexity by varying the number of N=2 and N=5 backstresses besides the consideration of the ratchetting behavior with N=5 and N=10 backstresses;
- The plasticity models and the determined temperature-dependent material properties are validated by means of in-phase and out-of-phase thermomechanical fatigue tests. The models lead to a very accurate description of the deformation behavior observed in isothermal low-cycle fatigue and thermomechanical fatigue experiments. However, not all compression tests are reasonably described;
- The ratchetting behavior is significantly overestimated by the pure Chaboche-type models incorporating no ratchetting term in the evolution of the Armstrong–Frederick hardening law. For N=5 backstresses, the ratchetting term leads to slight bending points in the stress–strain curves. Thus, N=10 backstresses should be applied to obtain smooth curves. However, the increase of the number of backstresses significantly increases the number of determinable material properties;
- The experience-based approach leads a priori to non-objective material properties, i.e., different persons would obtain miscellaneous material properties. Hence, future works should focus on quantitative analyses, e.g., by developing numerical optimization workflows, obtaining objective and physically-reasonable, temperature-dependent material properties for Chaboche-type models with multiple backstresses. These objective material properties can be used in material data spaces, e.g., to identify process–structure–properties between similar nickel-based superalloys.

## Figures and Tables

**Table 1:** Chemical composition of the investigated nickel-based superalloy IN100.

Co	Cr	Al	Ti	Mo	V	C	Zr	B	S	Fe	Cu	Si	Mn	Ni
14.8	9.0	5.7	4.7	3.0	0.8	0.16	0.06	0.014	0.003	<0.1	<0.1	<0.1	<0.1	base

## Data Availability

The data presented in this study are available on request from the corresponding author.

## Appendix A

The technical drawing of the fatigue specimens used in the cyclic experiments is shown in Figure A1.

For completeness, the temperature-dependent material properties of the pure Chaboche-type viscoplasticity models with N=2 backstresses are given in Table A1 and N=5 backstresses in Table A2. Table A3 summarizes the temperature-dependent material properties of the Chaboche-type viscoplasticity model combined with the Jiang ratchetting term with N=5 backstresses and Table A4 with N=10 backstresses, respectively.

**Table A1:** Temperature-dependent material properties of the Chaboche-type viscoplasticity model with N=2 backstresses for the nickel-based superalloy IN100.

Temperature	°C	300	750	850	950	1050
		Thermoelasticity				
Young’s modulus *E*	GPa	175	160	150	140	130
Thermal expansion coefficient $\alpha^{\mathrm{th}}$	$10^{-6}$/K	13.8801	15.2087	15.9233	16.8240	17.9132
		Viscosity				
Viscous reference stress *K*	MPa	50	300	400	450	500
Rate sensitivity exponent *n*	–	6.0	6.0	6.0	5.0	4.5
		Isotropic hardening				
Initial yield stress $R_{e}$	MPa	540	480	280	120	40
Saturation value $Q_{\infty }$	MPa	150	100	0	0	0
Hardening rate *b*	–	1.5	2.0	0	0	0
		Kinematic hardening—backstress 1				
Saturation value $C^{\left(1\right)}/\gamma^{\left(1\right)}$	MPa	165	165	165	140	80
Dynamic recovery $\gamma^{\left(1\right)}$	–	1200	1200	1200	1200	1200
Asymptotic steady state value $\phi_{\infty }^{\left(1\right)}$	–	1.0	1.0	1.0	1.2	1.5
Transition rate $\omega^{\left(1\right)}$	–	0	0	0	1.0	1.0
Static backstress recovery $R^{\left(1\right)}$	1/s	0	0.00025	0.00045	0.0006	0.0009
		Kinematic hardening—backstress 2				
Saturation value $C^{\left(2\right)}/\gamma^{\left(2\right)}$	MPa	330	330	330	280	160
Dynamic recovery $\gamma^{\left(2\right)}$	–	100	100	100	100	100
Asymptotic steady state value $\phi_{\infty }^{\left(2\right)}$	–	1.0	1.0	1.0	1.2	1.5
Transition rate $\omega^{\left(2\right)}$	–	0	0	0	1.0	1.0
Static backstress recovery $R^{\left(2\right)}$	1/s	0	0.00025	0.00045	0.0006	0.0009

**Table A2:** Temperature-dependent material properties of the Chaboche-type viscoplasticity model with N=5 backstresses for the nickel-based superalloy IN100.

Temperature	°C	300	750	850	950	1050
		Thermoelasticity				
Young’s modulus *E*	GPa	175	160	150	140	130
Thermal expansion coefficient $\alpha^{\mathrm{th}}$	$10^{-6}$/K	13.8801	15.2087	15.9233	16.8240	17.9132
		Viscosity				
Viscous reference stress *K*	MPa	50	300	400	450	500
Rate sensitivity exponent *n*	–	6.0	6.0	6.0	5.0	4.5
		Isotropic hardening				
Initial yield stress $R_{e}$	MPa	540	480	280	120	40
Saturation value $Q_{\infty }$	MPa	150	100	0	0	0
Hardening rate *b*	–	1.5	2.0	0	0	0
		Kinematic hardening—backstress 1				
Saturation value $C^{\left(1\right)}/\gamma^{\left(1\right)}$	MPa	80	80	80	60	40
Dynamic recovery $\gamma^{\left(1\right)}$	–	3000	3000	3000	3000	3000
Asymptotic steady state value $\phi_{\infty }^{\left(1\right)}$	–	1.0	1.0	1.0	1.2	1.5
Transition rate $\omega^{\left(1\right)}$	–	0	0	0	1.0	1.0
Static backstress recovery $R^{\left(1\right)}$	1/s	0	0.0004	0.0005	0.0006	0.0007
		Kinematic hardening—backstress 2				
Saturation value $C^{\left(2\right)}/\gamma^{\left(2\right)}$	MPa	60	60	60	50	35
Dynamic recovery $\gamma^{\left(2\right)}$	–	1000	1000	1000	1000	1000
Asymptotic steady state value $\phi_{\infty }^{\left(2\right)}$	–	1.0	1.0	1.0	1.2	1.5
Transition rate $\omega^{\left(2\right)}$	–	0	0	0	1.0	1.0
Static backstress recovery $R^{\left(2\right)}$	1/s	0	0.0004	0.0005	0.0006	0.0007
		Kinematic hardening—backstress 3				
Saturation value $C^{\left(3\right)}/\gamma^{\left(3\right)}$	MPa	120	120	120	120	75
Dynamic recovery $\gamma^{\left(3\right)}$	–	250	250	250	250	250
Asymptotic steady state value $\phi_{\infty }^{\left(3\right)}$	–	1.0	1.0	1.0	1.2	1.5
Transition rate $\omega^{\left(3\right)}$	–	0	0	0	1.0	1.0
Static backstress recovery $R^{\left(3\right)}$	1/s	0	0.0004	0.0005	0.0006	0.0007
		Kinematic hardening—backstress 4				
Saturation value $C^{\left(4\right)}/\gamma^{\left(4\right)}$	MPa	140	140	140	140	85
Dynamic recovery $\gamma^{\left(4\right)}$	–	50	50	50	50	50
Asymptotic steady state value $\phi_{\infty }^{\left(4\right)}$	–	1.0	1.0	1.0	1.2	1.5
Transition rate $\omega^{\left(4\right)}$	–	0	0	0	1.0	1.0
Static backstress recovery $R^{\left(4\right)}$	1/s	0	0.0004	0.0005	0.0006	0.0007
		Kinematic hardening—backstress 5				
Saturation value $C^{\left(5\right)}/\gamma^{\left(5\right)}$	MPa	180	180	180	165	115
Dynamic recovery $\gamma^{\left(5\right)}$	–	2.5	2.5	2.5	2.5	2.5
Asymptotic steady state value $\phi_{\infty }^{\left(5\right)}$	–	1.0	1.0	1.0	1.2	1.5
Transition rate $\omega^{\left(5\right)}$	–	0	0	0	1.0	1.0
Static backstress recovery $R^{\left(5\right)}$	1/s	0	0.0004	0.0005	0.0006	0.0007

**Table A3:** Temperature-dependent material properties of the Chaboche-type viscoplasticity model with Jiang ratchetting term with N=5 backstresses for the nickel-based superalloy IN100.

Temperature	°C	300	750	850	950	1050
		Thermoelasticity				
Young’s modulus *E*	GPa	175	160	150	140	130
Thermal expansion coefficient $\alpha^{\mathrm{th}}$	$10^{-6}$/K	13.8801	15.2087	15.9233	16.8240	17.9132
		Viscosity				
Viscous reference stress *K*	MPa	50	300	400	450	500
Rate sensitivity exponent *n*	–	6.0	6.0	6.0	5.0	4.5
		Isotropic hardening				
Initial yield stress $R_{e}$	MPa	540	480	280	120	40
Saturation value $Q_{\infty }$	MPa	150	100	0	0	0
Hardening rate *b*	–	1.5	2.0	0	0	0
		Kinematic hardening—backstress 1				
Saturation value $C^{\left(1\right)}/\gamma^{\left(1\right)}$	MPa	80	80	80	60	40
Dynamic recovery $\gamma^{\left(1\right)}$	–	3000	3000	3000	3000	3000
Asymptotic steady state value $\phi_{\infty }^{\left(1\right)}$	–	1.0	1.0	1.0	1.2	1.5
Transition rate $\omega^{\left(1\right)}$	–	0	0	0	1.0	1.0
Static backstress recovery $R^{\left(1\right)}$	1/s	0	0.0004	0.0005	0.0006	0.0007
Ratchetting exponent $\chi^{\left(1\right)}$	–	20	20	10	4	2
		Kinematic hardening—backstress 2				
Saturation value $C^{\left(2\right)}/\gamma^{\left(2\right)}$	MPa	60	60	60	50	35
Dynamic recovery $\gamma^{\left(2\right)}$	–	1000	1000	1000	1000	1000
Asymptotic steady state value $\phi_{\infty }^{\left(2\right)}$	–	1.0	1.0	1.0	1.2	1.5
Transition rate $\omega^{\left(2\right)}$	–	0	0	0	1.0	1.0
Static backstress recovery $R^{\left(2\right)}$	1/s	0	0.0004	0.0005	0.0006	0.0007
Ratchetting exponent $\chi^{\left(2\right)}$	–	25	25	15	7	3
		Kinematic hardening—backstress 3				
Saturation value $C^{\left(3\right)}/\gamma^{\left(3\right)}$	MPa	110	110	110	110	75
Dynamic recovery $\gamma^{\left(3\right)}$	–	200	200	200	200	200
Asymptotic steady state value $\phi_{\infty }^{\left(3\right)}$	–	1.0	1.0	1.0	1.2	1.5
Transition rate $\omega^{\left(3\right)}$	–	0	0	0	1.0	1.0
Static backstress recovery $R^{\left(3\right)}$	1/s	0	0.0004	0.0005	0.0006	0.0007
Ratchetting exponent $\chi^{\left(3\right)}$	–	50	50	25	10	5
		Kinematic hardening—backstress 4				
Saturation value $C^{\left(4\right)}/\gamma^{\left(4\right)}$	MPa	125	125	125	120	85
Dynamic recovery $\gamma^{\left(4\right)}$	–	25	25	25	25	25
Asymptotic steady state value $\phi_{\infty }^{\left(4\right)}$	–	1.0	1.0	1.0	1.2	1.5
Transition rate $\omega^{\left(4\right)}$	–	0	0	0	1.0	1.0
Static backstress recovery $R^{\left(4\right)}$	1/s	0	0.0004	0.0005	0.0006	0.0007
Ratchetting exponent $\chi^{\left(4\right)}$	–	100	100	50	15	7.5
		Kinematic hardening—backstress 5				
Saturation value $C^{\left(5\right)}/\gamma^{\left(5\right)}$	MPa	140	140	140	140	115
Dynamic recovery $\gamma^{\left(5\right)}$	–	2.5	2.5	2.5	2.5	2.5
Asymptotic steady state value $\phi_{\infty }^{\left(5\right)}$	–	1.0	1.0	1.0	1.2	1.5
Transition rate $\omega^{\left(5\right)}$	–	0	0	0	1.0	1.0
Static backstress recovery $R^{\left(5\right)}$	1/s	0	0.0004	0.0005	0.0006	0.0007
Ratchetting exponent $\chi^{\left(5\right)}$	–	200	200	100	20	10

**Table A4:** Temperature-dependent material properties of the Chaboche-type viscoplasticity model with Jiang ratchetting term with N=10 backstresses for the nickel-based superalloy IN100.

Temperature	°C	300	750	850	950	1050
		Thermoelasticity				
Young’s modulus *E*	GPa	175	160	150	140	130
Thermal expansion coefficient $\alpha^{\mathrm{th}}$	$10^{-6}$/K	13.8801	15.2087	15.9233	16.8240	17.9132
		Viscosity				
Viscous reference stress *K*	MPa	50	300	400	450	500
Rate sensitivity exponent *n*	–	6.0	6.0	6.0	5.0	4.5
		Isotropic hardening				
Initial yield stress $R_{e}$	MPa	540	480	280	120	40
Saturation value $Q_{\infty }$	MPa	150	100	0	0	0
Hardening rate *b*	–	1.5	2.0	0	0	0
		Kinematic hardening—backstress 1				
Saturation value $C^{\left(1\right)}/\gamma^{\left(1\right)}$	MPa	80	80	80	60	40
Dynamic recovery $\gamma^{\left(1\right)}$	–	3000	3000	3000	3000	3000
Asymptotic steady state value $\phi_{\infty }^{\left(1\right)}$	–	1.0	1.0	1.0	1.2	1.5
Transition rate $\omega^{\left(1\right)}$	–	0	0	0	1.0	1.0
Static backstress recovery $R^{\left(1\right)}$	1/s	0	0.0004	0.0007	0.0009	0.001
Ratchetting exponent $\chi^{\left(1\right)}$	–	20	20	10	4	2
		Kinematic hardening—backstress 2				
Saturation value $C^{\left(2\right)}/\gamma^{\left(2\right)}$	MPa	60	60	60	50	35
Dynamic recovery $\gamma^{\left(2\right)}$	–	1000	1000	1000	1000	1000
Asymptotic steady state value $\phi_{\infty }^{\left(2\right)}$	–	1.0	1.0	1.0	1.2	1.5
Transition rate $\omega^{\left(2\right)}$	–	0	0	0	1.0	1.0
Static backstress recovery $R^{\left(2\right)}$	1/s	0	0.0004	0.0007	0.0009	0.001
Ratchetting exponent $\chi^{\left(2\right)}$	–	25	25	12.5	6	3
		Kinematic hardening—backstress 3				
Saturation value $C^{\left(3\right)}/\gamma^{\left(3\right)}$	MPa	60	60	60	50	35
Dynamic recovery $\gamma^{\left(3\right)}$	–	400	400	400	400	400
Asymptotic steady state value $\phi_{\infty }^{\left(3\right)}$	–	1.0	1.0	1.0	1.2	1.5
Transition rate $\omega^{\left(3\right)}$	–	0	0	0	1.0	1.0
Static backstress recovery $R^{\left(3\right)}$	1/s	0	0.0004	0.0007	0.0009	0.001
Ratchetting exponent $\chi^{\left(3\right)}$	–	30	30	15	8	4
		Kinematic hardening—backstress 4				
Saturation value $C^{\left(4\right)}/\gamma^{\left(4\right)}$	MPa	40	40	40	40	30
Dynamic recovery $\gamma^{\left(4\right)}$	–	150	150	150	150	150
Asymptotic steady state value $\phi_{\infty }^{\left(4\right)}$	–	1.0	1.0	1.0	1.2	1.5
Transition rate $\omega^{\left(4\right)}$	–	0	0	0	1.0	1.0
Static backstress recovery $R^{\left(4\right)}$	1/s	0	0.0004	0.0007	0.0009	0.001
Ratchetting exponent $\chi^{\left(4\right)}$	–	50	50	25	10	5
		Kinematic hardening—backstress 5				
Saturation value $C^{\left(5\right)}/\gamma^{\left(5\right)}$	MPa	40	40	40	40	25
Dynamic recovery $\gamma^{\left(5\right)}$	–	75	75	75	75	75
Asymptotic steady state value $\phi_{\infty }^{\left(5\right)}$	–	1.0	1.0	1.0	1.2	1.5
Transition rate $\omega^{\left(5\right)}$	–	0	0	0	1.0	1.0
Static backstress recovery $R^{\left(5\right)}$	1/s	0	0.0004	0.0007	0.0009	0.001
Ratchetting exponent $\chi^{\left(5\right)}$	–	100	100	50	20	10
		Kinematic hardening—backstress 6				
Saturation value $C^{\left(6\right)}/\gamma^{\left(6\right)}$	MPa	40	40	40	40	25
Dynamic recovery $\gamma^{\left(6\right)}$	–	40	40	40	40	40
Asymptotic steady state value $\phi_{\infty }^{\left(6\right)}$	–	1.0	1.0	1.0	1.2	1.5
Transition rate $\omega^{\left(6\right)}$	–	0	0	0	1.0	1.0
Static backstress recovery $R^{\left(6\right)}$	1/s	0	0.0004	0.0007	0.0009	0.001
Ratchetting exponent $\chi^{\left(6\right)}$	–	100	100	50	20	10
		Kinematic hardening—backstress 7				
Saturation value $C^{\left(7\right)}/\gamma^{\left(7\right)}$	MPa	40	40	40	40	25
Dynamic recovery $\gamma^{\left(7\right)}$	–	20	20	20	20	20
Asymptotic steady state value $\phi_{\infty }^{\left(7\right)}$	–	1.0	1.0	1.0	1.2	1.5
Transition rate $\omega^{\left(7\right)}$	–	0	0	0	1.0	1.0
Static backstress recovery $R^{\left(7\right)}$	1/s	0	0.0004	0.0007	0.0009	0.001
Ratchetting exponent $\chi^{\left(7\right)}$	–	100	100	50	20	10
		Kinematic hardening—backstress 8				
Saturation value $C^{\left(8\right)}/\gamma^{\left(8\right)}$	MPa	40	40	40	40	25
Dynamic recovery $\gamma^{\left(8\right)}$	–	10	10	10	10	10
Asymptotic steady state value $\phi_{\infty }^{\left(8\right)}$	–	1.0	1.0	1.0	1.2	1.5
Transition rate $\omega^{\left(8\right)}$	–	0	0	0	1.0	1.0
Static backstress recovery $R^{\left(8\right)}$	1/s	0	0.0004	0.0007	0.0009	0.001
Ratchetting exponent $\chi^{\left(8\right)}$	–	200	200	100	40	20
		Kinematic hardening—backstress 9				
Saturation value $C^{\left(9\right)}/\gamma^{\left(9\right)}$	MPa	40	40	40	40	25
Dynamic recovery $\gamma^{\left(9\right)}$	–	5.0	5.0	5.0	5.0	5.0
Asymptotic steady state value $\phi_{\infty }^{\left(9\right)}$	–	1.0	1.0	1.0	1.2	1.5
Transition rate $\omega^{\left(9\right)}$	–	0	0	0	1.0	1.0
Static backstress recovery $R^{\left(9\right)}$	1/s	0	0.0004	0.0007	0.0009	0.001
Ratchetting exponent $\chi^{\left(9\right)}$	–	500	500	250	100	50
		Kinematic hardening—backstress 10				
Saturation value $C^{\left(10\right)}/\gamma^{\left(10\right)}$	MPa	60	60	60	60	40
Dynamic recovery $\gamma^{\left(10\right)}$	–	2.5	2.5	2.5	2.5	2.5
Asymptotic steady state value $\phi_{\infty }^{\left(10\right)}$	–	1.0	1.0	1.0	1.2	1.5
Transition rate $\omega^{\left(10\right)}$	–	0	0	0	1.0	1.0
Static backstress recovery $R^{\left(10\right)}$	1/s	0	0.0004	0.0007	0.0009	0.001
Ratchetting exponent $\chi^{\left(10\right)}$	–	1000	1000	500	200	100
